# Profiling the metabolic disorder and detection of colorectal cancer based on targeted amino acids metabolomics

**DOI:** 10.1186/s12967-023-04604-7

**Published:** 2023-11-17

**Authors:** Yang Yang, Zhipeng Wang, Xinxing Li, Jianfeng Lv, Renqian Zhong, Shouhong Gao, Feng Zhang, Wansheng Chen

**Affiliations:** 1 Department of Pharmacy, Second Affiliated Hospital of Naval Medical University, Shanghai, 200003 China; 2 Department of Pharmacy, the Affiliated Huaihai Hospital of Xuzhou Medical University / the 71st Group Army Hospital of CPLA Army, Xuzhou, 221004 Jiangsu China; 3 Department of General Surgery, Tongji Hospital, Tongji University, Shanghai, 200092 China; 4 Department of Pharmacy, Taixing People’s Hospital, Taixing, 225400 Jiangsu China; 5 Department of Laboratory Medicine, Second Affiliated Hospital of Naval Medical University, Shanghai, 200003 China

**Keywords:** Colorectal cancer, Amino acids, Targeted metabolomics, Diagnostic model, Transcriptome

## Abstract

**Background:**

The morbidity of cancer keeps growing worldwide, and among that, the colorectal cancer (CRC) has jumped to third. Existing early screening tests for CRC are limited. The aim of this study was to develop a diagnostic strategy for CRC by plasma metabolomics.

**Methods:**

A targeted amino acids metabolomics method was developed to quantify 32 plasma amino acids in 130 CRC patients and 216 healthy volunteers, to identify potential biomarkers for CRC, and an independent sample cohort comprising 116 CRC subjects, 33 precancerosiss patients and 195 healthy volunteers was further used to validate the diagnostic model. Amino acids-related genes were retrieved from Gene Expression Omnibus and Molecular Signatures Database and analyzed.

**Results:**

Three were chosen out of the 32 plasma amino acids examined. The tryptophan / sarcosine / glutamic acid -based receiver operating characteristic (ROC) curve showed the area under the curve (AUC) of 0.955 (specificity 83.3% and sensitivity 96.8%) for all participants, and the logistic regression model were used to distinguish between early stage (I and II) of CRC and precancerosiss patients, which showed superiority to the commonly used carcinoembryonic antigen. The GO and KEGG enrichment analysis proved many alterations in amino acids metabolic pathways in tumorigenesis.

**Conclusion:**

This altered plasma amino acid profile could effectively distinguish CRC patients from precancerosiss patients and healthy volunteers with high accuracy. Prognostic tests based on the tryptophan/sarcosine/glutamic acid biomarkers in the large population could assess the clinical significance of CRC early detection and intervention.

**Graphical Abstract:**

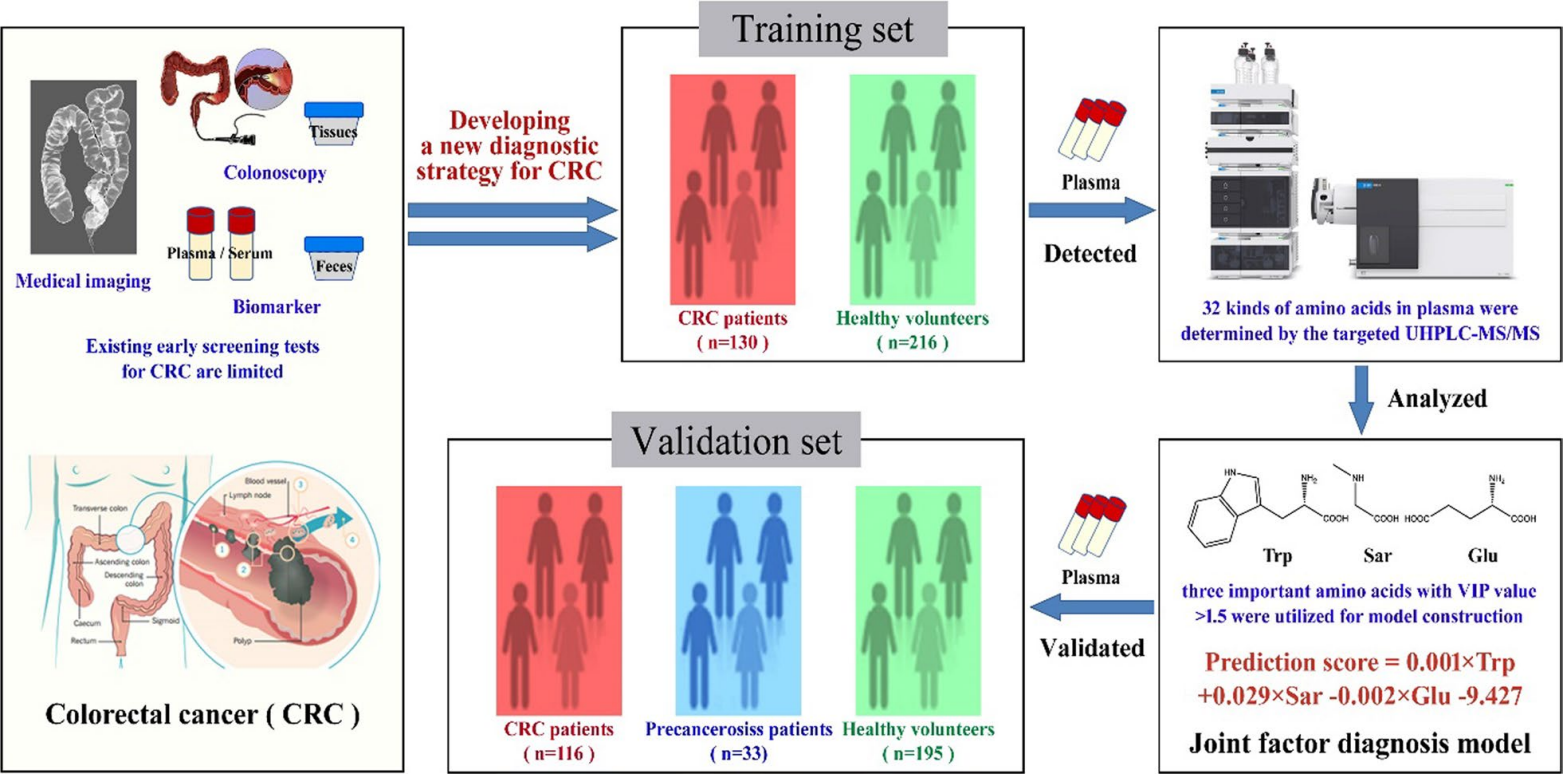

**Supplementary Information:**

The online version contains supplementary material available at 10.1186/s12967-023-04604-7.

## Highlights

1. The targeted amino acids metabolomics was applied to profile the alterations of amino acids in colorectal cancer.
2. Plasma amino acids profile shows capability of differentiating the colorectal cancer from the precancerosiss patients and healthy volunteers
3. A diagnostic model Y = 0.001×Trp +0.029×Sar-0.002×Glu-9.427 (unit: ng/mL) was developed and validated for colorectal cancer diagnosis.
4. Amino acids-related differentially expressed genes analysis supported the alterations of amino acids metabolic profile in tumorigenesis.

**Supplementary Information:**

The online version contains supplementary material available at 10.1186/s12967-023-04604-7.

## Introduction

The colorectal cancer (CRC) is one of the leading causes for cancer deaths worldwide, with more than 1.8 million people in 2018 [1, 2]. In China, CRC is the third-most frequently diagnosed cancer, with estimated 187 thousand deaths in 2015 [3]. Patients in the advanced-stage had significantly lower 5-year survival rate compared with those in the early-stage (stage IV, 5% versus stage I 95%) [4], but CRC was difficult to diagnose at early stage due to a lack of typical symptoms as well as specific and sensitive cancer biomarkers, for example, carcinoembryonic antigen (CEA). Therefore, early detection and intervention of CRC have been critical to prevent its negative impact. Unfortunately, clinical used methods for CRC screening, including endoscopy tests and fecal examinations, have some limitations [5]. The former is an invasive, inconvenient and expensive method, which is unfit for regular physical examination nationwide [6–8]; moreover, the available conventional endoscopic technique based on white-light imaging still holds a certain missing rate so that making it less efficient in early diagnosis [9]. The later helps to identify patients requiring endoscopic examination, but accuracy is far from optimal [10]. In addition, some non-coding RNAs, for example, the miR-211, miR-211, miR-122 have been reported as potential biomarkers for CRC diagnosis, but validations with large sample size are still to be completed [11–13]. Therefore, a convenient, economic method with high sensitivity and specificity is urgently required for the early detection of CRC.

Recently, the burgeoning omics field provides diverse high-throughput approaches for CRC blood sample-based biomarker discovery [14]. As the closest small molecular result of the final phenotype of the cancer, metabolomics is recognized as the robust and commonly used technology in cancer screening and diagnosis [15]. When compared with nontargeted approach, targeted metabolomics focusing on important known compounds or key pathways could achieve absolute quantification with high specificity and sensitivity, beneficial for the biomarker discovery and their translation into clinical application [16]. Indeed, recent studies have reported that there are many metabolite-related alternations including nucleotide metabolites, amino acids, bile acids, and short-chain fatty acids for the CRC patients. Amino acid metabolism, especially tryptophan metabolism and glutamate metabolism were the most of the altered metabolites through the analysis of nontargeted metabolomics [17–23]. Therefore, the targeted amino acids analysis could provide an insight into the current knowledge about the precise metabolome signatures, improving the reliability of CRC detection.

In this study, we used a targeted amino acids metabolomics method to profile 32 plasma amino acids in CRC patients and precancerosiss (PC) patients as well as healthy volunteers (HV) from three independent centers. A total of 690 plasma samples were analyzed, and differential amino acids were selected by means of orthogonal partial least squares-discriminant analysis (OPLS-DA) and logistic regression, and further validated, to identify the plasma amino acids as the potential diagnostic biomarkers for CRC. Besides, this study took advantage of transcriptomic data from GEO(Gene Expression Omnibus) database to depict the alterations of the amino acids between CRC tissue and adjacent normal tissue samples, which could consolidate the results of targeted amino acids metabolomics.

## Materials and methods

### Patients and sample collection

This is a multi-center, two sets and case–control study. The patients were enrolled in the Second Affiliated Hospital of Naval Medical University (Shanghai, China), the Affiliated Huaihai Hospital of Xuzhou Medical University (Xuzhou City, Jiangsu Province of China) and Taixing People’s Hospital (Taixing City, Jiangsu Province of China) between 2017 and 2018. The training set included 130 CRC patients and 216 HV, and the validation set was consisted of 116 CRC patients, 33 PC patients and 195 HV. The inclusion and exclusion criterions for CRC patients are as follows: (1) the patients with age ≥ 18 years old and clinically and histologically diagnosed with CRC were enrolled. (2) The patients who were diagnosed severe metabolic diseases, pregnancy, lactation, severe infection or other cancers were excluded. The protocol of this study was approved by the Medical Ethics Committee of the Second Affiliated Hospital of Naval Medical University, the Affiliated Huaihai Hospital of Xuzhou Medical University and Taixing People’s Hospital. All study participants signed the informed consent according to the institutional guidelines. The study was carried out in accordance with the 1964 Declaration of Helsinki and its later amendments or comparable ethical standards.

After the enrollment, the demographic characteristic data of all participants were documented, and the peripheral blood samples were collected in an EDTA-3 K tube from CRC patients in the morning on the day of surgery(food fasting overnight), and for HV and PC patients, the peripheral sample collection was completed after a food fasting overnight. All the blood samples were mixed gently and then subjected to a 2000 × g, 15 min centrifuge at 4 °C. The supernatant was aliquoted and transferred to CyroMax tubes, and all the plasma samples were stored in −80 °C until retrieval. The sample collection and process were accomplished within 1 h.

### Metabolites extraction and quantification analysis

The plasma sample pretreatment was developed based on protein precipitation. The three internal standards (IS) L-alanine-d4 (Ala-d4), L-methionine-d3 (Met-d3), and L-phenylalanine-d5 (Phe-d5) (400 ng/mL for each IS) were prepared freshly in acetonitrile, and a 50 μL aliquot of plasma sample was drawn and 150 μL acetonitrile (containing three IS) was added to precipitate the protein prior to a 3 min vortex-mixing and then the mixture was centrifuged at 19,060 × g, 4 °C for 15 min. The supernatant was injected directly to Agilent 1290-6460A ultra-high performance liquid chromatography tandem mass spectrometer (UHPLC-MS/MS) system for analysis.

The UHPLC-MS/MS quantitative analysis methods were developed in our group [24, 25]. On this basis, an targeted amino acids metabolomics method for determining the content of 32 amino acids in plasma was optimized [26] and 32 amino acids including glycine (Gly), L-alanine (Ala), L-valine (Val), L-lysine (Lys), L-leucine (Leu), L-isoleucine (Ile), L-gultamine (Gln), L-glutamic acid (Glu), L-methionine (Met), L-histidine (His), L-phenylalanine (Phe), L-arginine (Arg), L-tyrosine (Tyr), L-tryptophan (Trp), L-serine (Ser), L-proline (Pro), L-threonine (Thr), 5-oxo-L-proline (Opr), L-asparagine (Asn), L-ornithine (Orn), L-citrulline (Cit), L-cystine (Cyss), L-cysteine(Cys), 4-hydroxy-L-proline (Hpr), L-aspartic acid (Asp), asymmetric dimethylarginine (ADMA), symmetric dimethylarginine (SDMA), L-kynurenine (Kyn), 3-aminopropanoic acid (Apa), sarcosine (Sar), 3-amino-2-methylpropanoic acid (Amp), hippuric acid (Hia) were quantitatively analyzed in UHPLC-MS/MS system. The calibration standards were prepared using a phosphate buffer solution (1 × , namely 0.01 mol/L) as substituted matrix. The validation items including specificity, linearity, inter-day and intra-day accuracy and precision, extraction recovery and matrix effect, carry-over, stability, dilution effect were all assessed according to US Food and Drug Administration (FDA) guidance [27] and Chinese Pharmacopoeia (2015 Edition) [28], and this new method was proven to be sensitive, robust and efficient for the quantification of 32 amino acids in plasma.

### Transcriptomic data analysis

Two transcriptomic data sets(GSE164541 and GSE138202) from GEO database were retrieved. The counts of genes were normalized based on BaseMean to obtain the fold change and the negative binomial distribution test was carried out to dig out the differential genes (absolute fold change ≥ 2 and *P*-value < 0.05). The genes related to amino acids metabolism were downloaded from Molecular Signatures Database (AMINO_ACID_AND_DERIVATIVE_METABOLIC_PROCESS). All the analysis were completed using the OECloud tools based on R programming language (https://cloud.oebiotech.com). The enrichment analysis was carried out using Metascape V3.5.

### Statistical analysis

The metabolomics data were acquired and preprocessed using Agilent MassHunter workstation (version B.07.00). The student’s-*t* test was performed to compare the amino acids contents between different groups (IBM SPSS Statistics 21.0), and *P* < 0.05 was considered to be statistically significant. The multivariate statistical analysis was carried out using Umetrics Simca-p (version 14.1), and principal component analysis (PCA), OPLS-DA models, etc. were built to find the amino acids that contributed most to the model construction, and the amino acids with variable importance for the projection (VIP) value > 1 and false discovery rate (FDR) value < 0.05 would be selected for further diagnosis model construction. The model construction was developed in SPSS and the binary logistic regression was carried out to assess the biomarkers in differentiating the CRC.

## Results

### Population characteristics

The demographic data and clinical factors of the study cohort were summarized in Table 1. The patients in training set are consisted of 26 stage I, 39 stage II, 42 stage III, 14 stage IV and 9 unidentified stage CRC patients (112 adenocarcinoma, 6 tubular adenocarcinoma, 5 mucinous adenocarcinoma, 7 unknown), and in the validation set, 19 stage I, 43 stage II, 44 stage III, 7 stage IV and 3 unidentified stage CRC patients were enrolled. There were 13 and 10 CRC patients who had a neoadjuvant chemotherapy (5-FU-based regimen) in training set and validation set, respectively, and the samples of these patients were collected after a 4-week wash-out period to diminish the disturbance on the amino acids profile of drugs. To reduce the influence of age and gender between CRC patients and HV in training set, the distribution of age and gender were matched and no statistical differences were found (age, *P* = 0.188, student’s-*t* test; gender, *P* = 0.713, *χ*^2^ test), and no differences were found in the smoking status and alcohol consumption(*P* both > 0.05, *χ*^2^ test). The CEA and α-fetoprotein (AFP) values were retrieved as auxiliary diagnosis for all the patients, and in the training set, the CEA of CRC patients was higher than the HV (*P* = 0.000, Mann–Whitney *U* test), while the AFP was lower in CRC patients (*P* < 0.01, Mann–Whitney *U* test), and in the validation set, the CEA (*P* = 0.000, Mann–Whitney *U* test) and AFP (*P* = 0.000, Mann–Whitney *U* test) were higher in the CRC patients compared with the HV. In the validation set, 33 PC patients including 20 polyps, 7 inflammatory colitis, 1 intestinal obstruction and 5 mixed hemorrhoids were recruited because higher risk for CRC.

**Table 1:** Demographic and clinical characteristics of study cohorts

Items	Training set		Validation set		
	CRC	HV	CRC	PC	HV
n	130	216	116	33	195
Gender (n, male/female)	72/58	124/92	83/33	22/11	89/106
Age (mean ± SD)	59.68 ± 11.53	58.07 ± 10.65	61.38 ± 12.51	50.94 ± 12.18	48.02 ± 14.87
Range	28–89	24–83	26–89	24 ~ 72	24 ~ 76
CEA (median, range)	3.22, 0.49 ~ 2056	0.70, 0.08 ~ 8.96	2.28, 0.94–277.5	2.15, 0.23–6.52	0.43, 0.06–7.87
AFP (median, range)	2.54, 1.04 ~ 8.00	2.98, 0.15 ~ 11.75	3.03, 1.06–7.95	3.15, 2.13–6.36	2.53, 0.06–5.82
Presampling chemotherapy(n)	13		10		
CRC stages(n)					
I	26		19		
II	39		43		
III	42		44		
IV	14		7		
NA	9		3		
Pathological pattern					
Adenocarcinoma	112				
Tubular adenocarcinoma	6				
Mucinous adenocarcinoma	5				
Unknown	7				
PC category(n)					
Polyp				20	
Inflammatory colitis				7	
Intestinal obstruction				1	
Mixed hemorrhoids				5	
Smoker(n)	26	35			
Alcohol consumption(> 100 g/day)	23	30			

### Amino acids profile in plasma samples

**Fig. 1:**
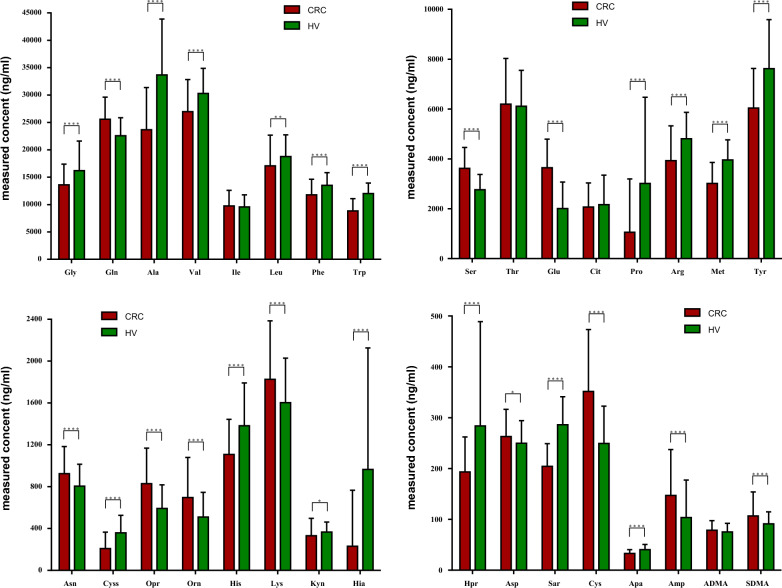
Comparative contents of 32 amino acids between CRC and HV plasma samples from training set (^*^P < 0.05, ^**^P < 0.01, ^***^P < 0.001, ^****^P < 0.0001)

In the training set, 346 plasma samples from 130 CRC patients and 216 HV were analyzed, and concentrations of 32 amino acids were quantified in these samples (Additional file 1: Fig. S1A). In the training set, the concentrations of 17 amino acids including Gly, Ala, Val, Leu, Phe, Trp, Pro, Arg, Met, Tyr, Cyss, His, Kyn, Hia, Hpr, Sar, and Apa significantly decreased in CRC patients compared with that in HV (*P* < 0.05, student’s-*t* test), while Gln, Ser, Glu, Asn, Opr, Orn, Lys, Asp, Cys, Amp, and SDMA obviously increased in CRC (all *P* < 0.05, student’s-*t* test), and four amino acids Ile, Thr, Cit, and ADMA didn’t show any differences between CRC patients and HV (Fig. 1). The other 33 plasma samples from PC patients were added to the validation set, and totally 344 plasma samples consisting of 116 CRC patients, 33 PC patients and 195 HV were measured using the same method, and concentrations of 32 amino acids were quantified in these samples (Additional file 1: Fig. S1B). Most of the amino acids showed similar variation trends in training and validation sets between CRC and HV, except for Arg, Orn and Asp, which present inverse variation trends between CRC patients and HV. For the PC patients, the concentrations of all amino acids are comparable to the CRC patients and HV (Additional file 1: Fig. S1C). In a word, we found that most of the amino acids differed between CRC patients and HV, which may reveal the metabolic disorders of amino acids in CRC patients.

### Identification of amino acids associated with CRC

**Fig. 2:**
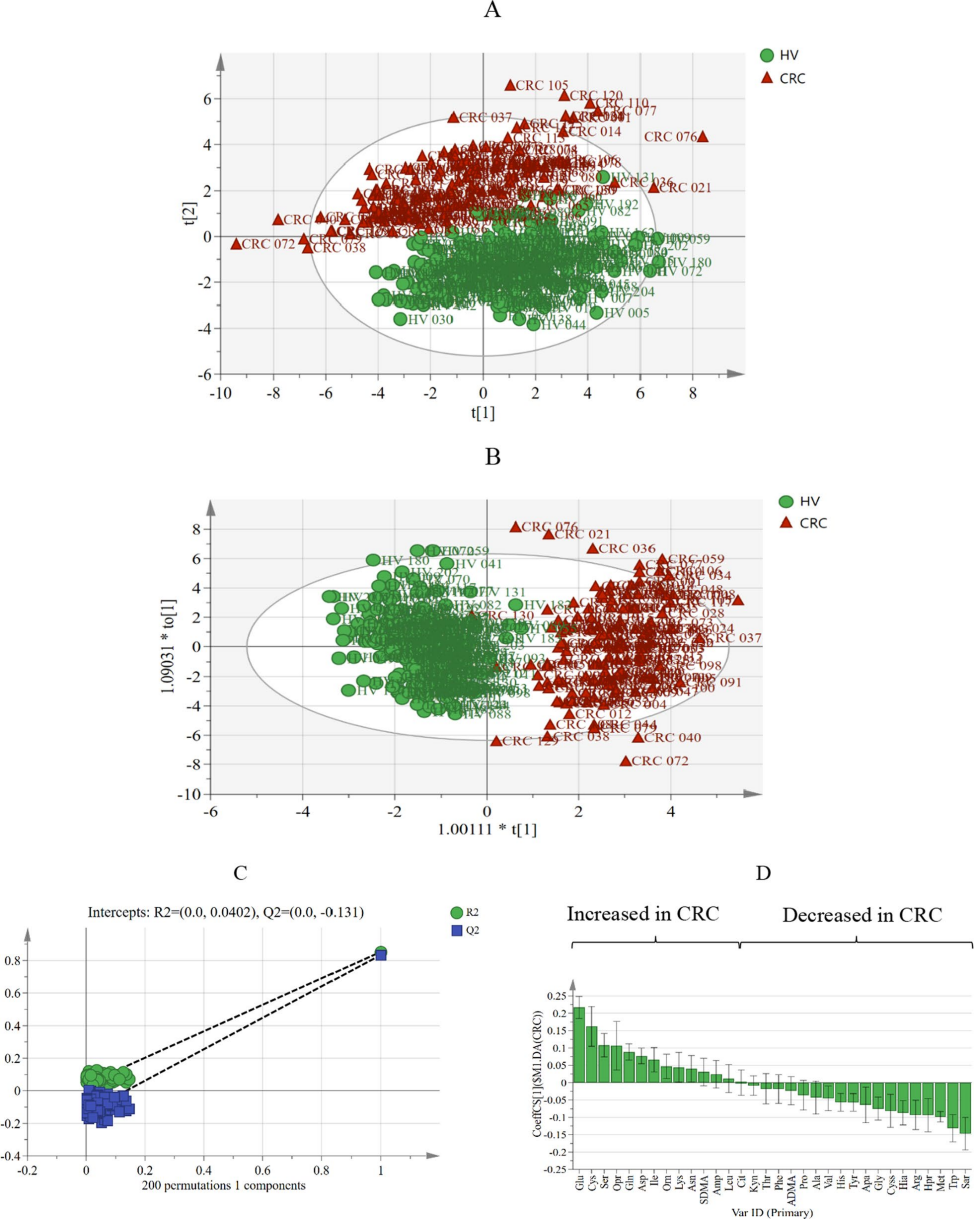
Multivariate statistical analysis for discrimination model construction and differential compounds screening. **A**: Score scatter plot for PCA model of amino acids metabolic profile in plasma samples of training set presented a separation trend between CRC and HV[R2X(cum) = 0.499, Q2(cum) = 0.292]. **B**: Score scatter plot for OPLS-DA model of amino acids metabolic profile in CRC and HV plasma samples of training set, and good separation was obtained [R2X(cum) = 0.414, R2Y(cum) = 0.853, Q2(cum) = 0.834]. **C**: The plot of response permutation testing proved the reliability of OPLS-DA model (n = 200, HV). **D**: Coefficient column plot for OPLS-DA of CRC vs. matched control, illustrating changes of amino acids in CRC occurrence

**Fig. 3:**
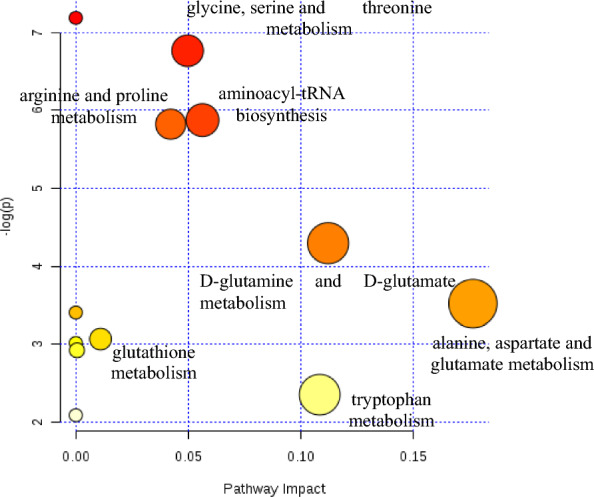
Metabolic pathway enrichment analysis of differential amino acids. The featured amino acids were enriched in KEGG database, and alanine, aspartate and glutamate metabolism, D-glutamine and D-glutamate metabolism, tryptophan metabolism, glycine, serine and threonine metabolism, aminoacyl-tRNA biosynthesis, arginine and proline metabolism pathways showed most alterations in CRC occurrence

A hierarchical cluster analysis was preformed to visualize the comprehensive differences and correlations of amino acids between CRC patients and HV (Additional file 1: Fig. S2), and the results presented differences of amino acids profile between CRC and HV; and to find the amino acids that had most discrepancy, the 32 amino acids profile in the training set were further analyzed based on a multivariate statistical method. A PCA model was established, but the results showed no specific clustering in relation to clinical factors. The HV clustered tighter than CRC patients and a separation trend could be seen between CRC patients and HV [R2X(cum) = 0.499, Q2(cum) = 0.292] (Fig. 2A and Additional file 1: Fig. S3A). In an OPLS-DA model, the CRC patients and HV were clearly separated [R2X(cum) = 0.414, R2Y(cum) = 0.853, Q2(cum) = 0.834] (Fig. 2B and Additional file 1: Fig. S3B). Cross-validation analysis of variance (CV-ANOVA, n = 200) *P*-value was less than 0.001 and the intercept of permutation test Q2 was −0.131, which demonstrate that this model is stable and non-random (Fig. 2C). Then, coefficient column plot and loading scatter plot of amino acid metabolic profiles (Fig. 2D and Additional file 1: Fig. S4) were built and 14 out of 32 amino acids including Trp, Sar, Glu, Ser, Met, Ala, Cys, Cyss, Tyr, Opr, Apa, Gln, Hia, and Arg passed the filter procedure (VIP value > 1 and FDR value < 0.05). The results were shown in Table 2. The amino acids with VIP value > 1.5 were selected and Trp, Sar and Glu were incorporated to a metabolic pathway analysis in Kyoto Encyclopedia of Genes and Genomes (KEGG) database, and the results found that they were associated with alanine, aspartate and glutamate metabolism, D-glutamine and D-glutamate metabolism, tryptophan metabolism, glycine, serine and threonine metabolism, aminoacyl-tRNA biosynthesis, arginine and proline metabolism, etc. (Fig. 3).

**Table 2:** VIP values and t-test results of amino acids

Variable	VIP value	Results of t tests (*p* value)
Trp	1.70147	*P* < 0.0001
Sar	1.69872	*P* < 0.0001
Glu	1.62941	*P* < 0.0001
Ser	1.42921	*P* < 0.0001
Met	1.41820	*P* < 0.0001
Ala	1.40305	*P* < 0.0001
Cys	1.35692	*P* < 0.0001
Cyss	1.17065	*P* < 0.0001
Tyr	1.16002	*P* < 0.0001
Opr	1.09176	*P* < 0.0001
Apa	1.05048	*P* < 0.0001
Gln	1.04221	*P* < 0.0001
Hia	1.03793	*P* < 0.0001
Arg	1.02590	*P* < 0.0001

### Construction and validation of diagnostic model

As an obvious different amino acids profile was found between CRC patients and HV, we made an effort to construct a discriminant model for the CRC diagnosis. Three amino acids with VIP > 1.5 including Trp, Sar and Glu were finally chosen for the model construction based on binary logistics regression, and finally the joint factor diagnosis model was developed as follows:

Joint factor prediction score = 0.001 × Trp + 0.029 × Sar−0.002 × Glu−9.427 (unit: ng/mL).

**Fig. 4:**
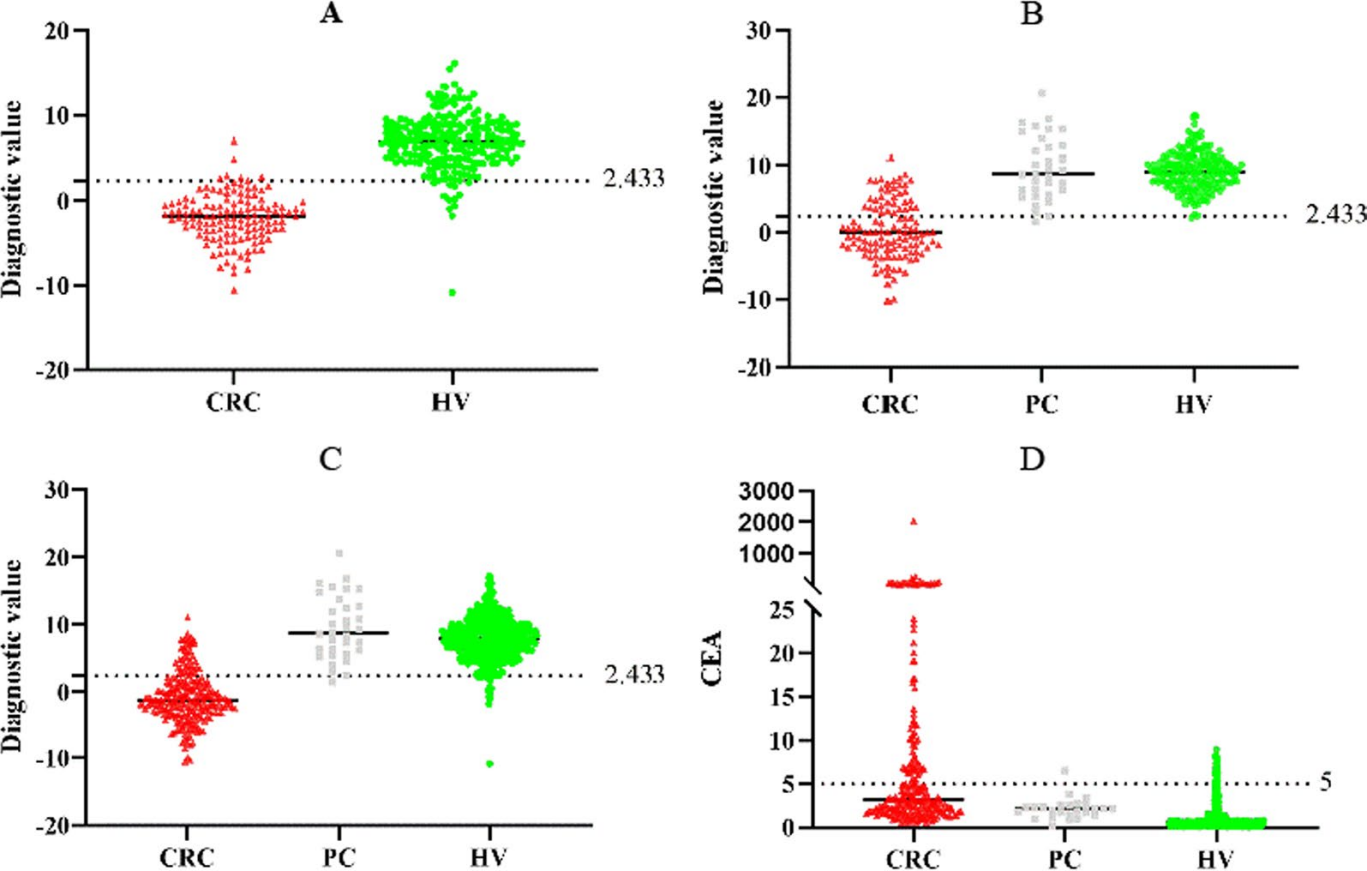
Diagnostic model construction and assessment. **A**: Construction of diagnostic model in training set showed entirely differentiation of CRC patients and HV based on three amino acids (Sensitivity: 95.4%; Specificity: 94.4%). **B**: Testing the diagnostic model in validation set comprising of CRC patients, PC patients and HV shows good ability to differentiate the CRC (Sensitivity: 70.0%; Specificity: 98.7%). **C**: Testing the diagnostic model in all participates showed high sensitivity and specificity (Sensitivity: 83.3%; Specificity: 96.8%). **D**: Validating the CEA for CRC diagnosis with clinical cutoff value 5 ng/mL results poor sensitivity for CRC screening (Sensitivity: 31.7%; Specificity: 92.7%)

**Fig. 5:**
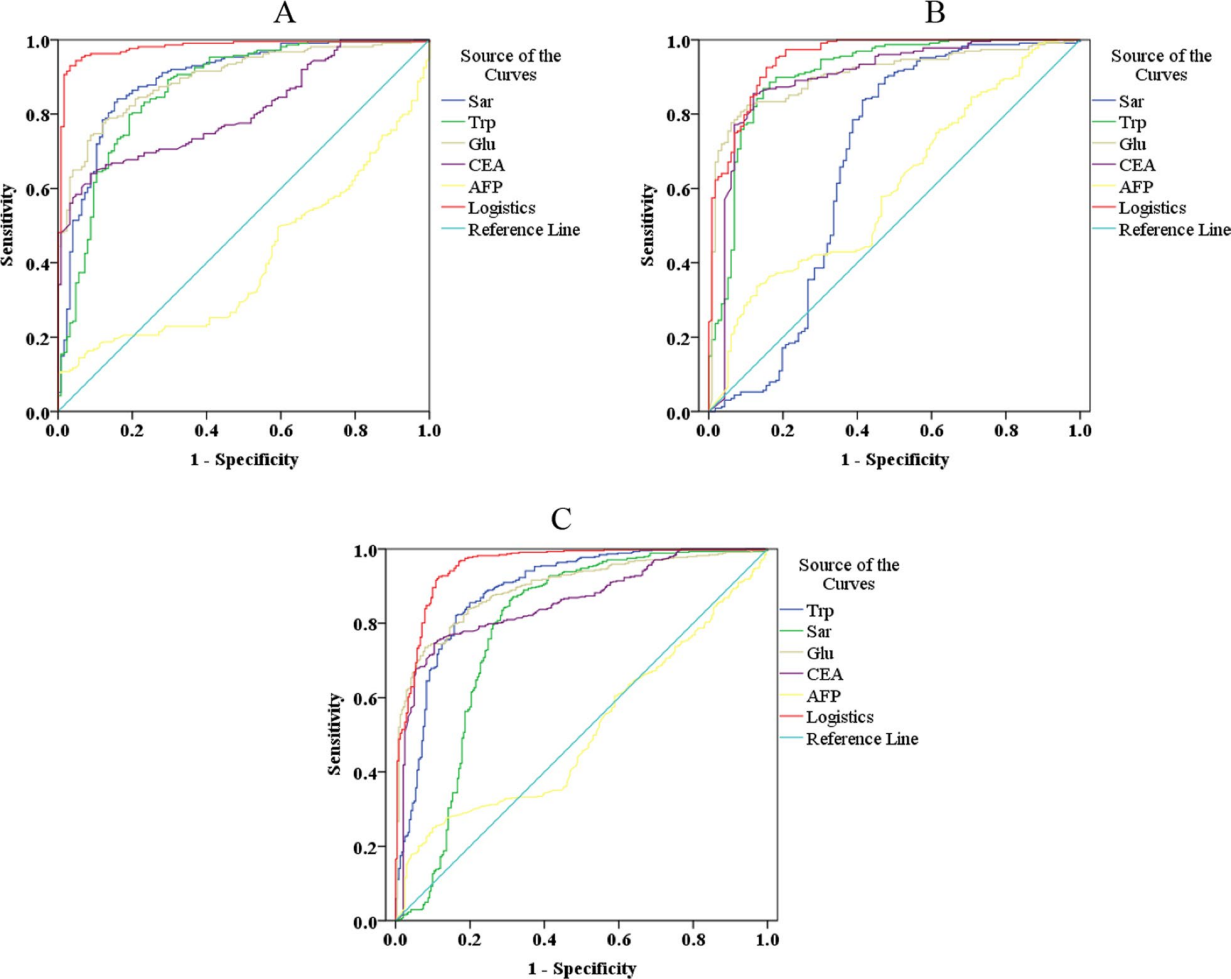
Diagnostic model construction and assessment. **A**: The ROC curves of joint factor diagnostic model, single amino acid, CEA and AFP in CRC and HV plasma samples of training set. **B**: The ROC curves of joint factor diagnostic model, single amino acid, CEA and AFP in CRC and control plasma samples of validation set. **C**: The ROC curves of joint factor diagnostic model, single amino acid, CEA and AFP in CRC and control plasma samples of all participants

The logistics regression model parameters were shown in Additional file 1: Table S1. The area under the curve (AUC) showed the diagnosis performance of single amino acid, CEA, AFP, and the joint factor diagnosis model in the training set (Fig. 4A and Fig. 5), and the results prove an excellent diagnosis performance of the joint factor diagnosis model (AUC = 0.980, 95% CI 0.965 ~ 0.994), and the AUC = 0.861 (95% CI 0.818 ~ 0.903), 0.888 (95% CI 0.852 ~ 0.925), 0.896 (95% CI 0.864 ~ 0.928), 0.809 (95% CI 0.764 ~ 0.853), and 0.402 (95% CI 0.341 ~ 0.462) for Trp, Sar, Glu, CEA, and AFP, respectively. The results were shown in Additional file 1: Table S2. The cutoff value was set as 2.433 when the Youden’s index reached the maximum. The specificity was 0.944 and the sensitivity was 0.954 of this diagnostic model in the training set, and the measured value lower than 2.433 could be discriminated as CRC (Table 3). The model was then validated in an independent set, and the AUC = 0.951 (95%CI 0.928 ~ 0.974) for the model, which was higher than Trp (AUC = 0.909, 95% CI 0.872 ~ 0.945), Sar (AUC = 0.666, 95% CI 0.594 ~ 0.739) and Glu (AUC = 0.905, 95% CI 0.872 ~ 0.938), and the sensitivity and specificity were 70.0% and 98.7%, respectively. The results were shown in Fig. 4B. Furthermore, we combined the training set and the validation set as one cohort, and our diagnosis model was finally assessed in this cohort. The AUC = 0.955 (95% CI 0.965 ~ 0.994) with 83.3% specificity and 96.8% sensitivity (Fig. 4C). Our diagnosis model was able to discriminate the CRC patients from HV and PC patients, and the sensitivity and specificity showed much advantage than conventional CEA (the cutoff value set as 5 ng/mL), which gave 31.7% sensitivity and 92.7% specificity (Fig. 4D and Additional file 1: Tables S3, S4).

**Table 3:** The efficacy evaluation of diagnostic factors

Variable	The value of cut off	Sensitivity	1-Specificity	Max Youden's index
Trp	10,509.57 ng/ml	0.808	0.204	0.604
Sar	237.09 ng/ml	0.823	0.162	0.661
Glu	2492.72 ng/ml	0.869	0.218	0.652
Joint factor diagnostic model	2.433	0.954	0.056	0.898

### Detecting stages of CRC and early-stage diagnosis

Furthermore, We assumed the stage I and stage II CRC as early stage, and stage III and stage IV as advanced stage, and the stage-related amino acids profile was analyzed by a new PCA model. The CRC samples distributed evenly in the PCA model and no stage-related clusters were found [R2X(cum) = 0.376, Q2(cum) = 0.239] (Additional file 1: Fig. S5A), and similar results were shown in OPLS-DA model [R2X(cum) = 0.344, Q2(cum) = 0.089] (Additional file 1: Fig. S5B). The amino acids profile couldn’t recognize the CRC stages. To assess the diagnosis power of this model for the early stage CRC, we combined the stage I and stage II CRC in the training set and the same stages CRC in the validation set, and 128 early stage CRC were obtained. The sensitivity of CEA was 28.1% while our diagnosis model showed an 85.42% sensitivity, demonstrating an obvious superiority in early stage CRC diagnosis of this model.

### Tumorigenesis disturbed the amino acids profile

**Fig. 6:**
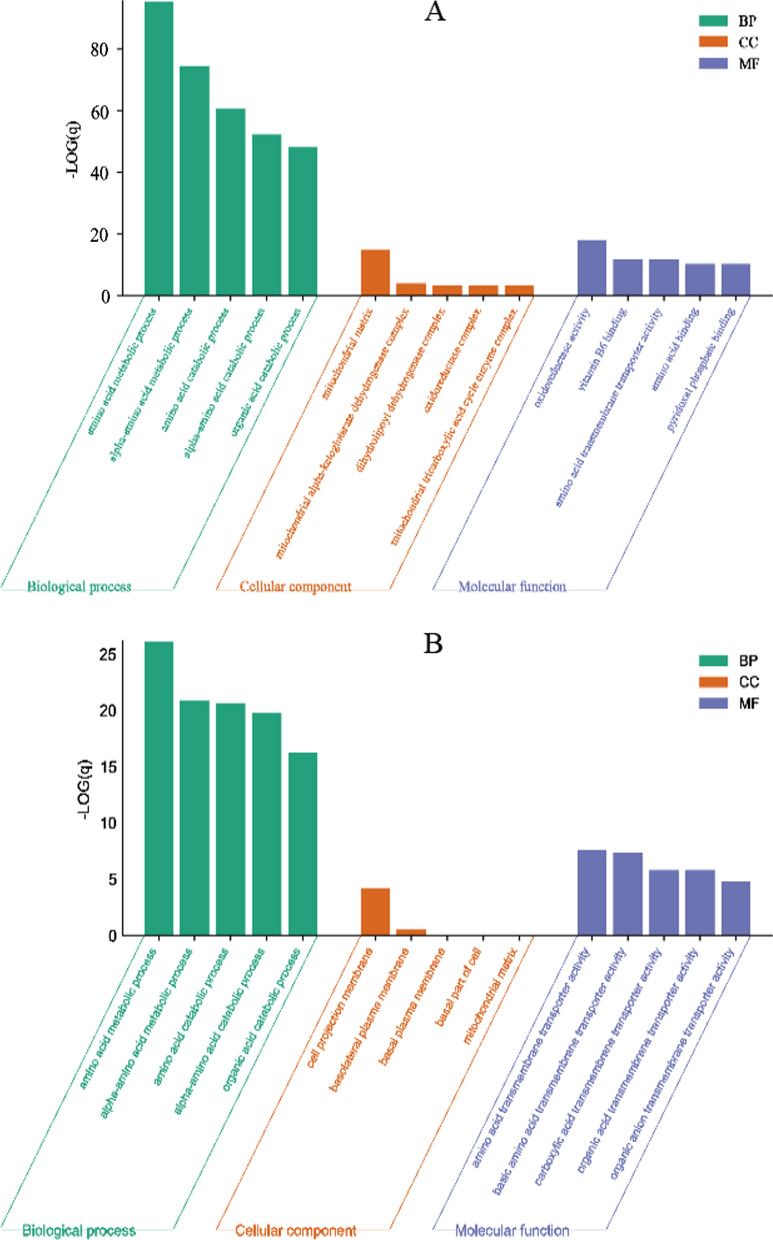
The GO enrichment analysis were carried out for differentially expressed amino acids-related genes, and the top 5 terms in BP, CC, and MF were shown. A: differentially expressed amino acids-related genes based on dataset GSE164541; B: differentially expressed amino acids-related genes based on dataset GSE138202

Two datasets(GSE164541 and GSE138202) including 5 and 8 paired tumor and adjacent normal tissue samples, respectively, were analyzed and in the GSE 164541, 2172 and 2046 significantly upregulated and downregulated genes were found in tumorigenesis, respectively, and in GSE138202, 4477 and 3791 significantly upregulated and downregulated genes were dug out in tumorigenesis with the threshold q-value < 0.05 and − 1 > log FC > 1, respectively(Additional file 1: Fig. S6A and B). Totally 101 amino acids-related genes from Molecular Signatures Database were downloaded and combined with the two GSE datasets. Subsequently, 94 and 38 amino acids-related genes that showed significantly differences were subjected to enrichment analysis, and the results(GSE164541) proved that the top 5 BP(biological process) terms included amino acid metabolic process, alpha-amino acid metabolic process, amino acid catabolic process, alpha-amino acid catabolic process, organic acid catabolic process, and the top 5 MF(molecular function) terms were oxidoreductase activity, vitamin B6 binding, amino acid transmembrane transporter activity, amino acid binding and pyridoxal phosphate binding; and the data from GSE 138202 showed similar results in top 5 BP terms, while its presented amino acid transmembrane transporter activity, basic amino acid transmembrane transporter activity, carboxylic acid transmembrane transporter activity, organic acid transmembrane transporter activity, organic anion transmembrane transporter activity as top 5 MF terms(Fig. 6A, B). On the other hand, the KEGG pathway analysis indicated similar pathways, for instance, the alanine, aspartate and glutamate metabolism, cysteine and methionine metabolism, biosynthesis of amino acids, arginine and proline metabolism, etc., were significantly enriched (Additional file 1: Fig. S7). These results may support the alterations of amino acids’ metabolic profile in blood induced by tumorigenesis and its capability in CRC diagnosis.

## Discussion

Available evidences have shown that dramatic metabolic changes are crucial for initiation and progression of CRC, and the metabolite biomarkers varied based upon the stage of CRC, and methods of analysis. However, there were still many commonly identified metabolites among the different reports [29]. Specifically, amino acid metabolism has been frequently characterized as significantly altered in CRC pathogenesis [30], For example, some of the amino acids, also known as glycogenic amino acids, including the Gly, Ala, Ser, Val, Asn, Asp, Gln, Glu, Arg, Cys, Met, Pro and His, could be transformed into pyruvic acid, α-ketoglutaric acid, succinic acid and oxaloacetic acid through deamination and transamination and then diverted into the tricarboxylate (TCA) cycle to supply the energy, and Leu and Lys, which were called ketogenic amino acids, could be catabolized into acetylcoenzyme A and acetic acid and then replenished the TCA cycle, and the Thr, Ile, Phe, Tyr and Trp showed dual roles in ketogenesis and glycogenesis [31]. Furthermore, the Asp was the substitute for pyrimidine synthesis and Gly, Gln and Asp could provide the nitrogen groups for purine synthesis [32]. These reported alterations of amino acids metabolism were supported by the results of transcriptomic data in this study.

In this study, a diagnostic model was constructed based on three amino acids with an excellent sensitivity (0.954) and specificity (0.944), and then validated in an independent group which was consisted of 116 CRC patients, 33 PC patients and 195 HV. The results of validation process presented a good sensitivity (70.0%) and specificity (98.7%), and a better sensitivity (83.3%) and specificity (96.8%) were gained when the training set and validation set were combined. Besides, this model has an obvious superiority over CEA in CRC early diagnosis (stage I and stage II CRC). Although the amino acids profile could not distinguish the early stage and advanced stage of CRC, Opr, Kyn and Trp showed significant increase trend along with the CRC progression. A study reported that the concentrations of some sphingomyelins, phosphatidylcholines and two amino acids Cit and His in plasma were related to CRC progression [33] and another study showed a lower Gln, His and Ala and higher Gly in serum from advanced stage CRC [34]. These data successfully elucidated that CRC was accompanied by the presence or absence of specific amino acids or altered amino acid metabolism.

Generally, the diagnostic model was generated by a combination of three metabolites (tryptophan / sarcosine / glutamic acid), capable of differentiating between CRC patients and HV. Our research found a significant decrease of Trp in CRC compared with HV, and this result was supported by the gene expression change (Additional file 1: Fig. S7A) and many other studies [35–38]. The lower level of Trp in CRC patients’ plasma probably related to the enhanced uptake of Trp by cancer cell, which was drove by proto-oncogene MYC gene [39]. Sar, a non-essential amino acid, is an intermediate and byproduct in Gly synthesis and degradation, and our study found that Gly, Ser and Thr metabolism pathway has remarkably altered in cancer (Additional file 1: Fig. S7A). A study reported a decline of Sar in serum from CRC patients, which was in accordance with our results [17]. Elevated Sar was found to be associated with CRC risk [40], and in a chemical-induced colorectal cancer in mice, too [41]. Furthermore, higher level of Sar was delineated as a differential metabolite in prostate cancer and played a role in prostate cancer progression, and this increase was shown in prostate cancer tissue, plasma and urine [42]. Gln and its metabolite Glu both contribute as nitrogen and/or carbon sources (Additional file 1: Fig. S7B) to the biosynthesis of important cellular constituents [43, 44]. The elevated level of Glu in CRC patients in our study may attribute to the disturbance of fatty acids and nucleotides biosynthesis which Gln participates and lead to an increased Glu level. This result was further supported by another study, which detected a 1.8-fold increase of Glu in CRC serum [45].

The first shortcoming of our research is that only a targeted amino acids metabolomics method was utilized to profile the metabolic disorders of CRC patients and HV, which may lead to some loss of metabolic features. Combining the untargeted and targeted metabolomics methods possess a higher opportunity to clarify the metabolome alterations of CRC and develop the diagnostic tool for CRC. Besides, it is important to take into account the influence imposed by the microbiota in the generation and consumption of metabolites, so that integrated analysis of the faecal metagenome and serum metabolome was a considerable method for the CRC detection [46]. Thirdly, although our results have been validated in an independent cohort, the number of included subjects was relatively small. Of note, further studies are required to enhance the capacity to distinguish different stages of CRC, especially the early stages, and improve the prediction of prognosis; moreover, a larger sample size could favor the understanding of clinical significance of metabolites despite of some demographic features, such as gender, age, BMI and smoking status. Future studies are still needed to explain the relevance of the changes in amino acid profiles in the etiology-and-pathomechanism of CRC.

**Fig. 7:**
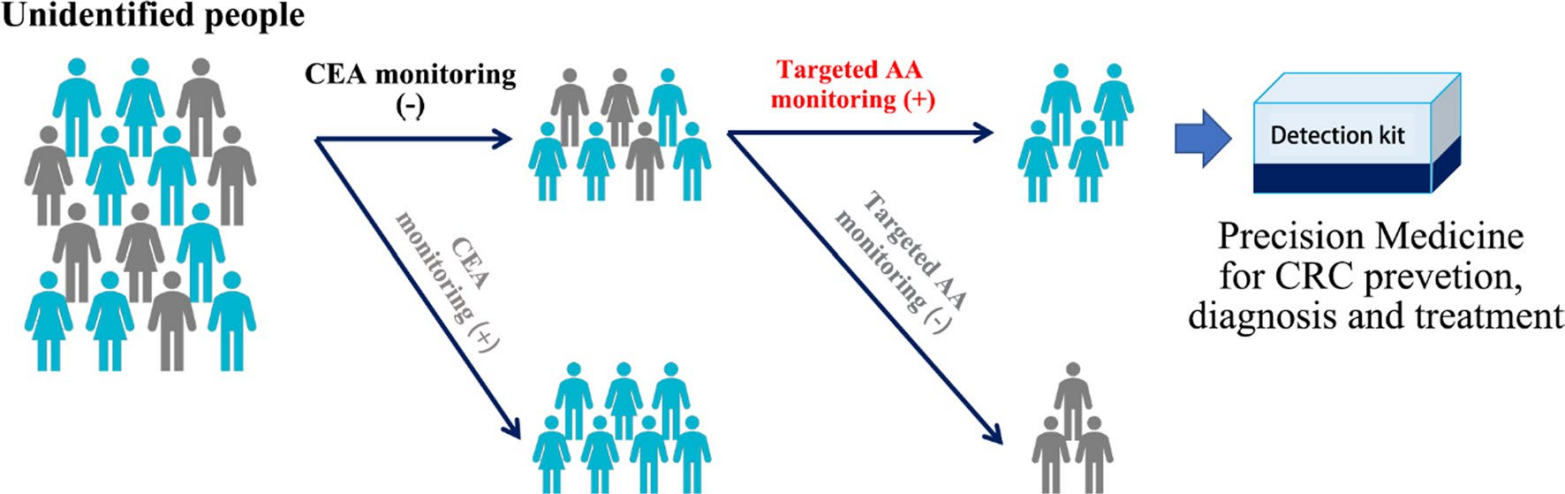
The diagnostic model based on amino acids monitoring improves the CRC detection

In conclusion, we applied a targeted amino acids metabolomics method to measure 32 amino acids in plasma samples from CRC patients, PC patients and HV, and profiled the metabolic disorder of amino acids in CRC patients, and then differentially expressed genes of CRC tissue sample were retrieved from online database and analyzed, and the results proved the alterations of amino acids-related genes expressions in tumorigenesis. A diagnostic model was constructed using Trp, Glu and Sar, and this model was validated in an independent group and excellent sensitivity and specificity were obtained. This model may be a better tool for the CRC early screen with higher sensitivity and specificity and non-invasive characteristic other than the CEA and colonoscopy (Fig. 7).

### Supplementary Information

**Additional file 1: Figure S1A.** Total ion current graphs of 32 amino acids in plasma samples from training set. a. CRC patient; b. HV. **Figure S1B.** Total ion current graphs of 32 amino acids in plasma samples from validation set. a. CRC patient; b. PC patient; c. HV. **Figure S1C.** Contents of 32 amino acids in plasma samples of validation set. **Figure S2**. A hierarchical cluster analysis to visualize the comprehensive differences and correlations of amino acids between CRC and HV. **Figure S3A.** Plot cumulative R2X(cum) and Q2(cum) of components for PCA model of amino acids metabolic profiles in plasma samples from training set. **Figure S3B.** Polt cumulative R2X(cum) and Q2(cum) of components for OPLS-DA model of amino acids metabolic profiles in CRC and HV plasma samples from training set. **Figure S4.** Loading scatter plot of amino acid metabolic profiles in CRC and HV plasma samples from training set. **Figure S5A.** Score scatter plot for PCA model of amino acids profiles in early and advanced stage CRC patients’ plasma samples. **Figure S5B**. Score scatter plot for OPLS-DA model of amino acids profiles in early and advanced stage CRC patients’ plasma samples. **Figure S6A.** Volcano plot of differentially expressed genes in dataset GSE164541. **Figure S6B**. Volcano plot of differentially expressed genes in dataset GSE 138202. **Figure S7.** Summarized pathway changes in tumorigenesis based on The KEGG pathway analysis. A: pathway enrichment based on GSE164541; B pathway enrichment based on GSE138202. **Table S1.** Logistics regression model parameters. **Table S2.** Areas under the curve of diagnostic factors. **Table S3.** The value of joint factor diagnostic model in the diagnosis of CRC. **Table S4.** The value of CEA in the diagnosis of CRC.

## Data Availability

The data of this study are available upon request.

## Abbreviations

ADMA: Asymmetric dimethylarginine
AFP: α-Fetoprotein
Ala: L-alanine
Ala-d4: L-alanine-d4
Amp: 3-Amino-2-methylpropanoic acid
Apa: 3-Aminopropanoic acid
Arg: L-arginine
Asn: L-asparagine
Asp: L-aspartic acid
AUC: Area under the curve
CEA: Carcinoembryonic antigen
Cit: L-citrulline
CRC: Colorectal cancer
Cys: L-cysteine
Cyss: L-cystine
FDA: Food and Drug Administration
FDR: False discovery rate
Gln: L-glutamine
Glu: L-glutamic acid
Gly: Glycine
Hia: Hippuric acid
His: L-histidine
Hpr: 4-Hydroxy-L-proline
HV: Healthy volunteer
Ile: L-isoleucine
IS: Internal standard
Kyn: L-kynurenine
Leu: L-leucine
Lys: L-lysine
Met: L-methionine
Met-d3: L-methionine-d3
OPLS-DA: Orthogonal partial least squares-discriminant analysis
Opr: 5-Oxo-L-proline
Orn: L-ornithine
PC: Precancerosiss
PCA: Principal component analysis
Phe: L-phenylalanine
Phe-d5: L-phenylalanine-d5
Pro: L-proline
ROC: Receiver-operating characteristic
Sar: Sarcosine
SDMA: Symmetric dimethylarginine
Ser: L-serine
TCA: Tricarboxylic acid
Thr: L-threonine
Trp: L-tryptophan
Tyr: L-tyrosine
UHPLC-MS/MS: Ultra-high performance liquid chromatography tandem mass spectrometry
Val: L-valine
VIP: Variable importance for the projection
GEO: Gene expression omnibus
KEGG: Kyoto encyclopedia of genes and genomes
