# Supplementary material for: Profiling the metabolic disorder and detection of colorectal cancer based on targeted amino acids metabolomics
Source: J Transl Med. 2023 Nov 17;21:824. doi: 10.1186/s12967-023-04604-7 (PMC10655464; doi:10.1186/s12967-023-04604-7)
Supplement: Supplementary file 1 — Additional file 1: Figure S1A. Total ion current graphs of 32 amino acids in plasma samples from training set. a. CRC patient; b. HV. Figure S1B. Total ion current graphs of 32 amino acids in plasma samples from validation set. a. CRC patient; b. PC patient; c. HV. Figure S1C. Contents of 32 amino acids in plasma samples of validation set. Figure S2. A hierarchical cluster analysis to visualize the comprehensive differences and correlations of amino acids between CRC and HV. Figure S3A. Plot cumulative R2X(cum) and Q2(cum) of components for PCA model of amino acids metabolic profiles in plasma samples from training set. Figure S3B. Polt cumulative R2X(cum) and Q2(cum) of components for OPLS-DA model of amino acids metabolic profiles in CRC and HV plasma samples from training set. Figure S4. Loading scatter plot of amino acid metabolic profiles in CRC and HV plasma samples from training set. Figure S5A. Score scatter plot for PCA model of amino acids profiles in early and advanced stage CRC patients’ plasma samples. Figure S5B. Score scatter plot for OPLS-DA model of amino acids profiles in early and advanced stage CRC patients’ plasma samples. Figure S6A. Volcano plot of differentially expressed genes in dataset GSE164541. Figure S6B. Volcano plot of differentially expressed genes in dataset GSE 138202. Figure S7. Summarized pathway changes in tumorigenesis based on The KEGG pathway analysis. A: pathway enrichment based on GSE164541; B pathway enrichment based on GSE138202. Table S1. Logistics regression model parameters. Table S2. Areas under the curve of diagnostic factors. Table S3. The value of joint factor diagnostic model in the diagnosis of CRC. Table S4. The value of CEA in the diagnosis of CRC. [file 12967_2023_4604_MOESM1_ESM.docx]

**Additional file**

**Profiling the metabolic disorder and detection of colorectal cancer based on targeted amino acids metabolomics**

Yang Yang^1,2,5#^, Zhipeng Wang^1#^, Xinxing Li^3^, Jianfeng Lv^4^, Renqian Zhong^5^,
Shouhong Gao^1*^, Feng Zhang^1*^, Wansheng Chen^1*^

(1. Department of Pharmacy, the Second Affiliated Hospital of Naval Medical University of CPLA, Shanghai 200003, China; 2. Department of Pharmacy, the Affiliated Huaihai Hospital of Xuzhou Medical University / the 71st Group Army Hospital of CPLA Army, Jiangsu Xuzhou 221004, China; 3. Department of General Surgery, Tongji Hospital, Tongji University, Shanghai 200092, China; 4. Department of Pharmacy, Taixing People’s Hospital, Jiangsu Taixing 225400, China; 5. Department of Laboratory Diagnostics, the Second Affiliated Hospital of Naval Medical University of CPLA, Shanghai 200003, China )

^#^ These authors contributed equally to this work.

Yang Yang: newyyang@163.com (ORCID: https://orcid.org/0000-0001-5618-3940); Zhipeng Wang: wangzhipeng@smmu.edu.cn (ORCID: https://orcid.org/0000-0002-7964-9288)

Correspondence should be addressed to Shouhong Gao: gaoshouhong@smmu.edu.cn, Feng Zhang: fengzhang@smmu.edu.cn, and Wansheng Chen: chenwansheng@smmu.edu.cn

| 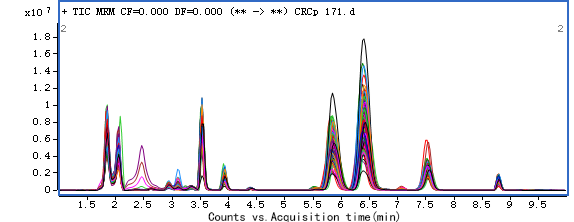  **a** |
| --- |
| 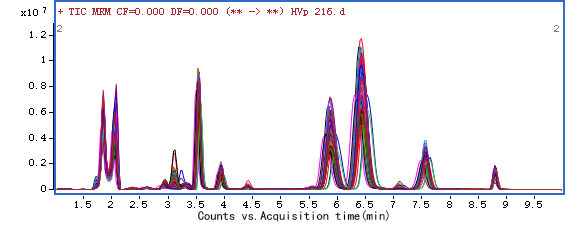  **b** |

**Additional Fig. S1A** Total ion current graphs of 32 amino acids in plasma samples from training set. a. CRC patient; b. HV

| 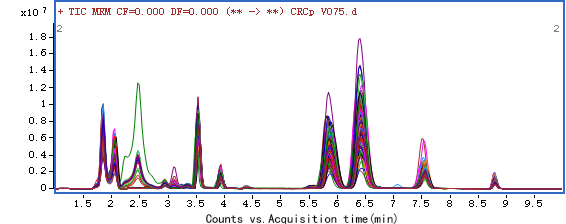  **a** |
| --- |
| 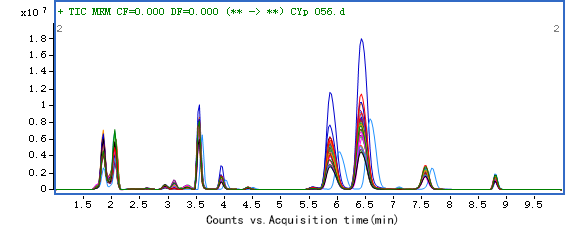  **b** |
| 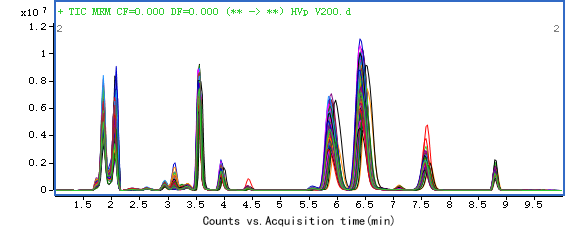  **c** |

**Additional Fig. S1B** Total ion current graphs of 32 amino acids in plasma samples from validation set. a. CRC patient; b. PC patient; c. HV

|  |  |
| --- | --- |
|  |  |
|  |  |

**Additional Fig. S1C** Contents of 32 amino acids in plasma samples of validation set


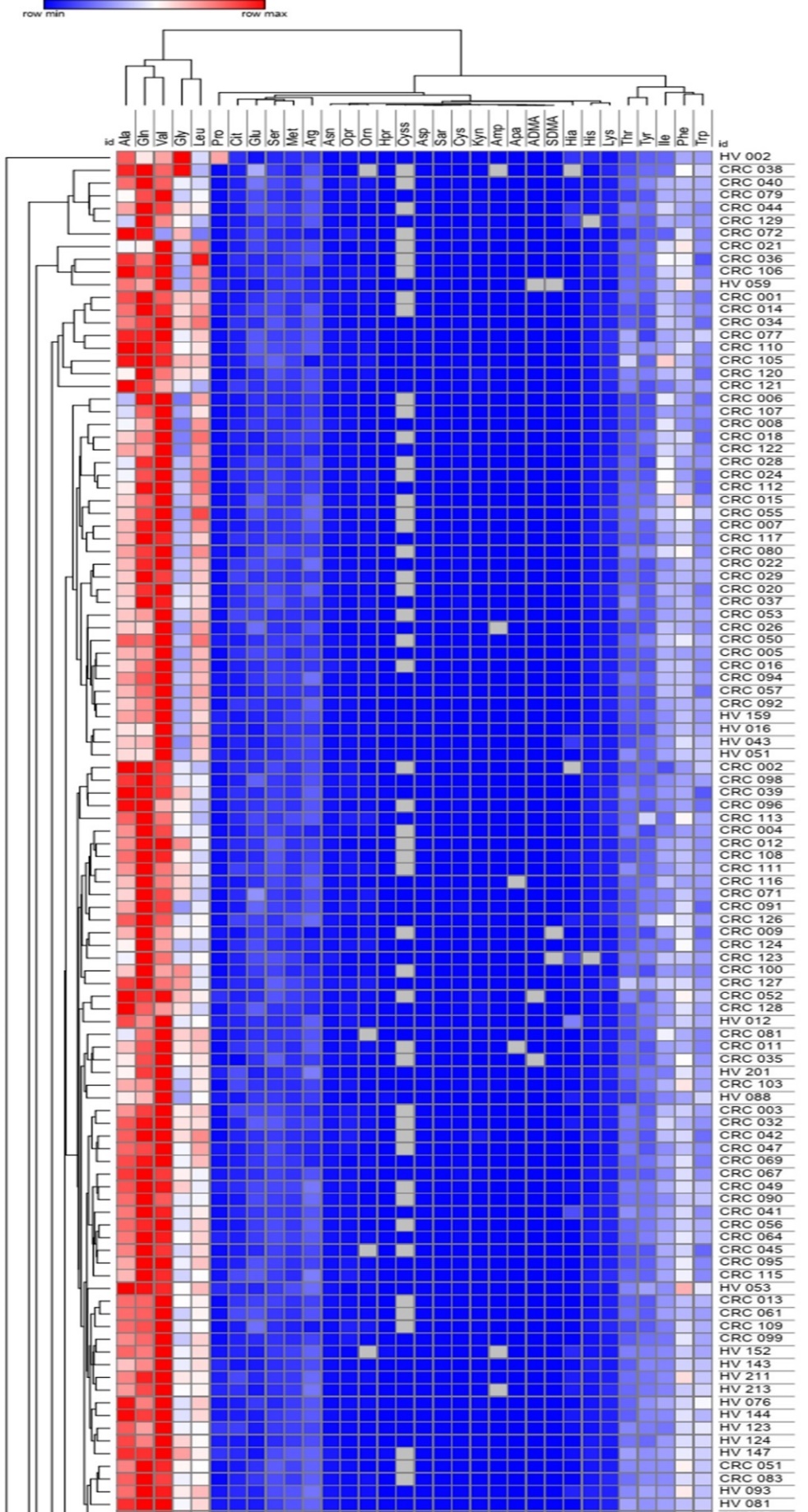


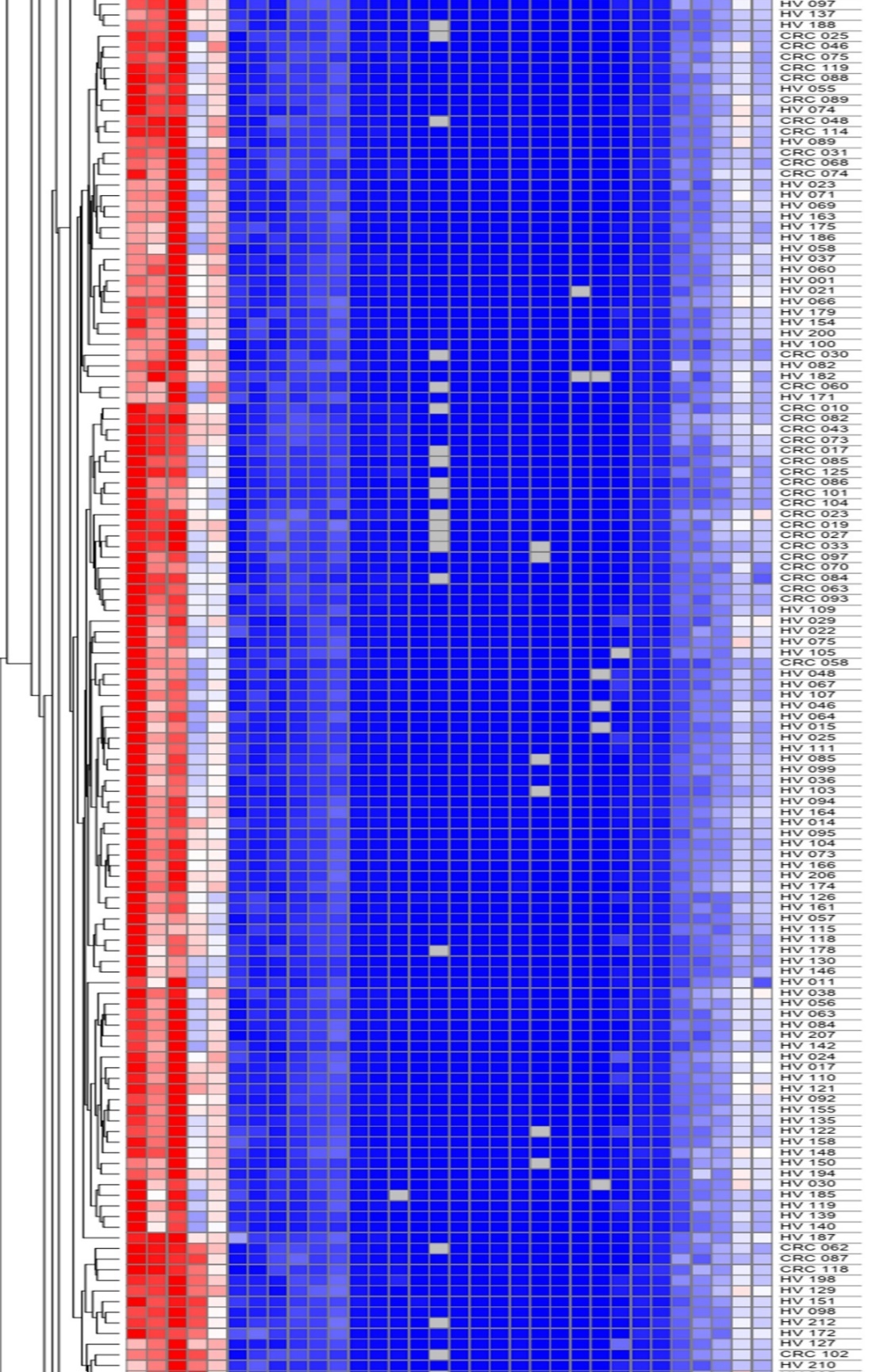


**Additional Fig. S2A (continued)**


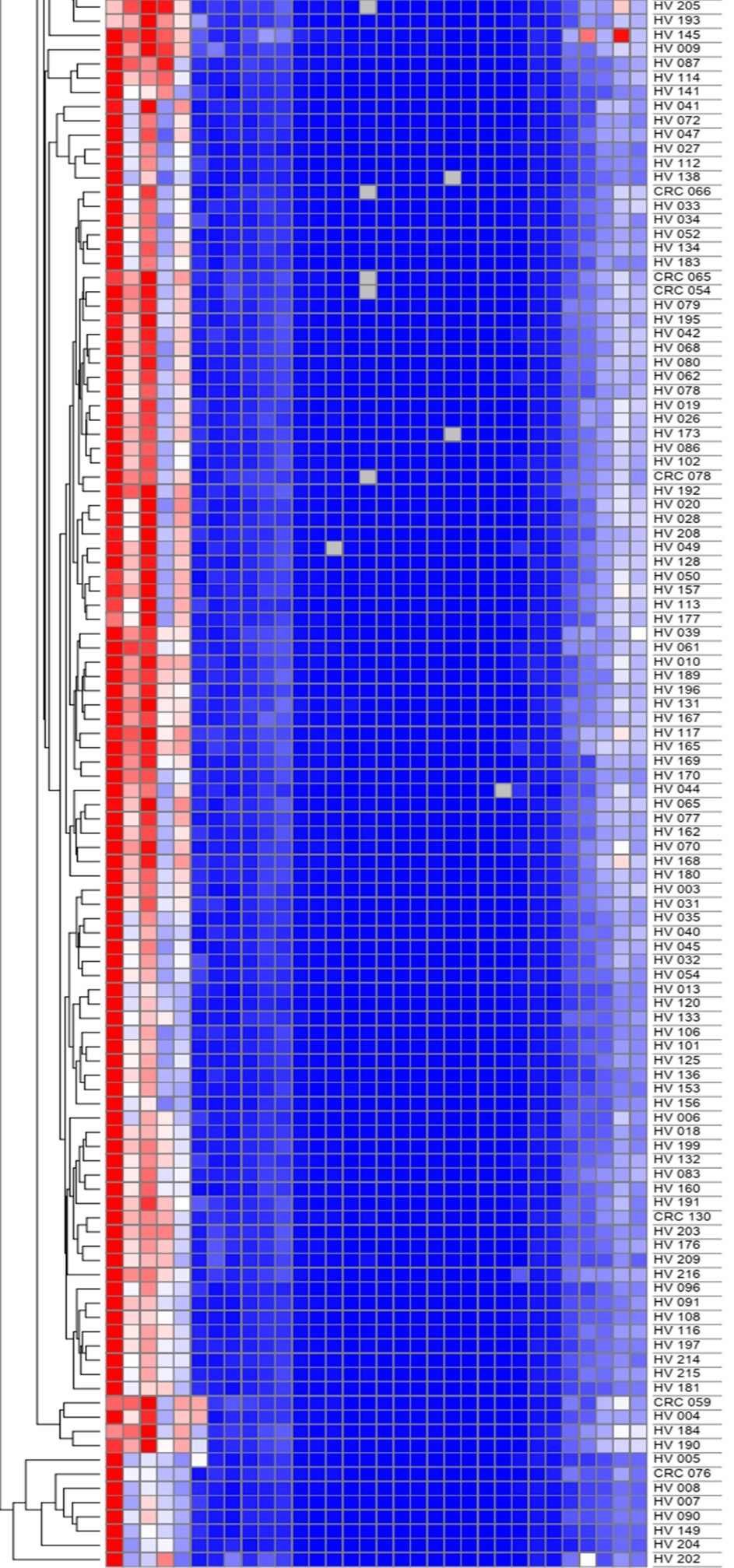


**Additional Fig. S2A (continued)**

**Additional Fig. S2** A hierarchical cluster analysis to visualize the comprehensive differences and correlations of amino acids between CRC and HV.


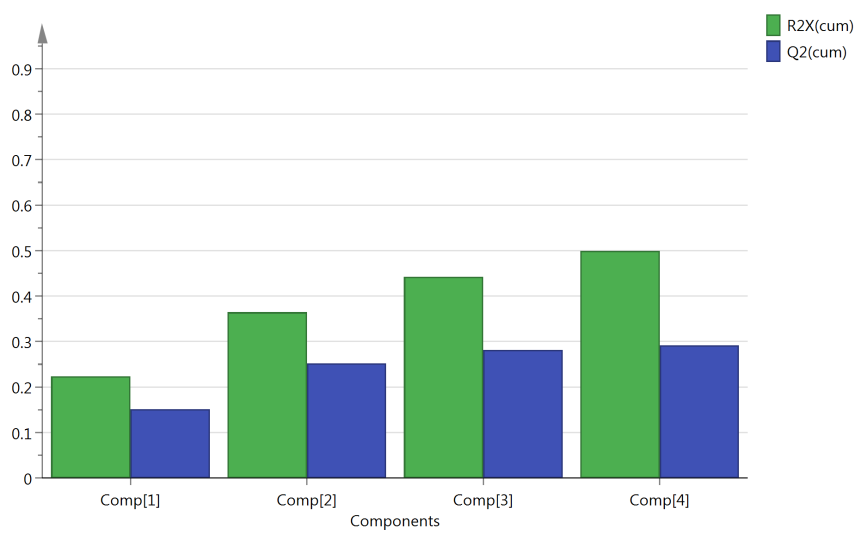


**Additional Fig. S3A** Plot cumulative R2X(cum) and Q2(cum) of components for PCA model of amino acid metabolic profiles in plasma samples from training set.

**
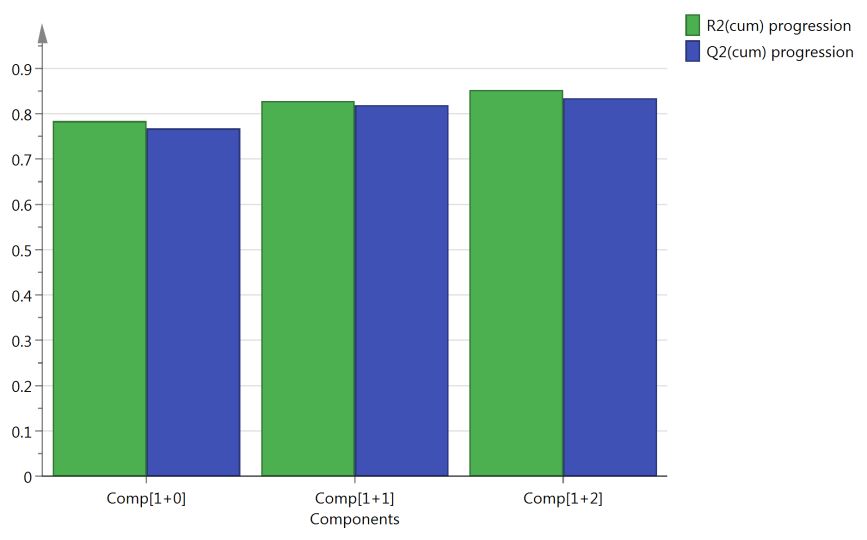
**

**Additional Fig. S3B** Polt cumulative R2X(cum) and Q2(cum) of components for OPLS-DA model of amino acid metabolic profiles in CRC and HV plasma samples from training set.


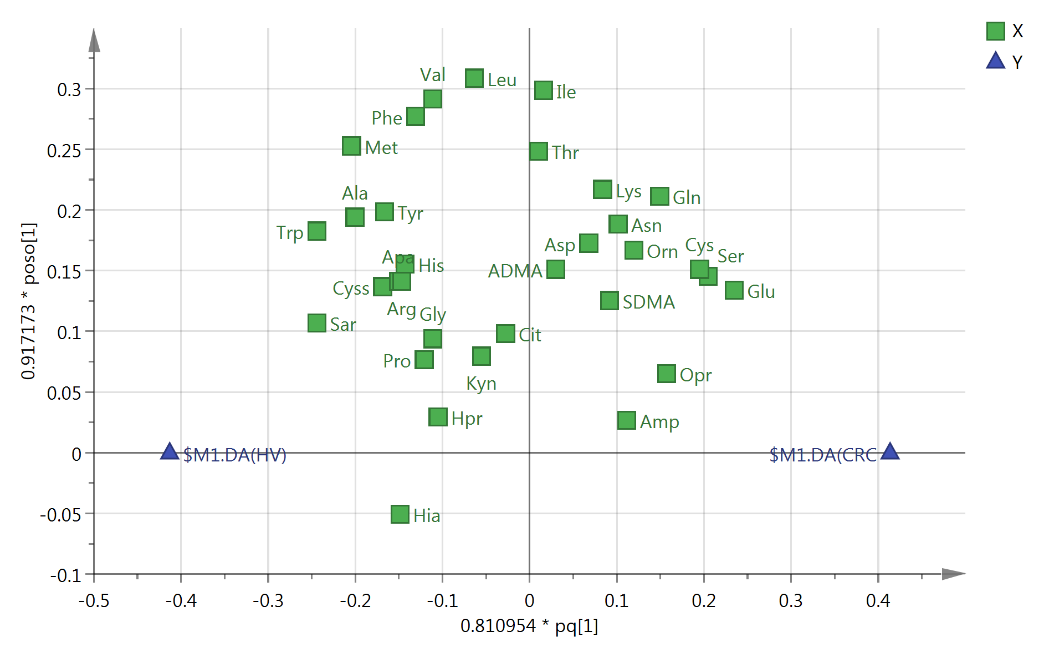


**Additional Fig. S4** Loading scatter plot of amino acid metabolic profiles in CRC and HV plasma samples from training set.


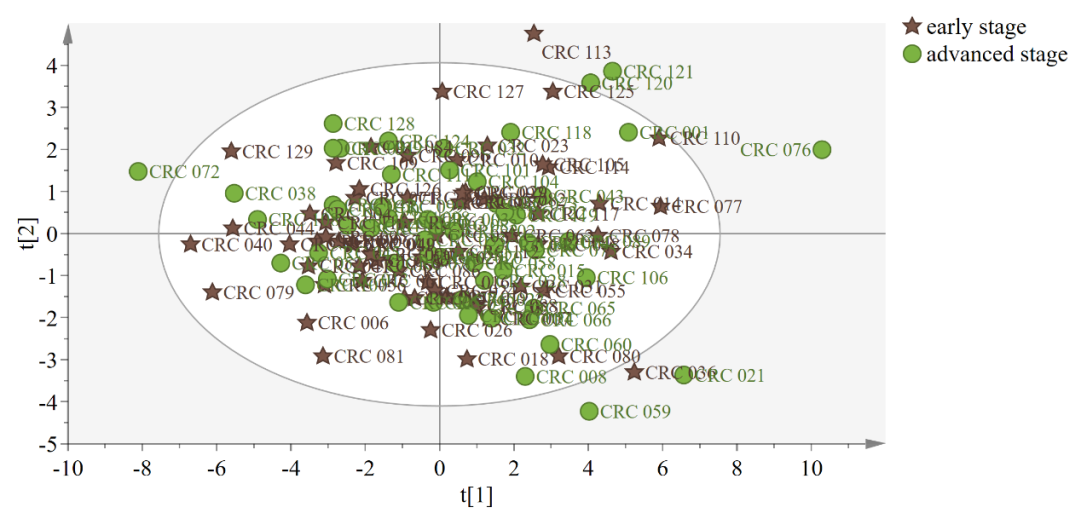


**Additional Fig. S5A** Score scatter plot for PCA model of amino acids profiles in early and advanced stage CRC patients’ plasma samples.


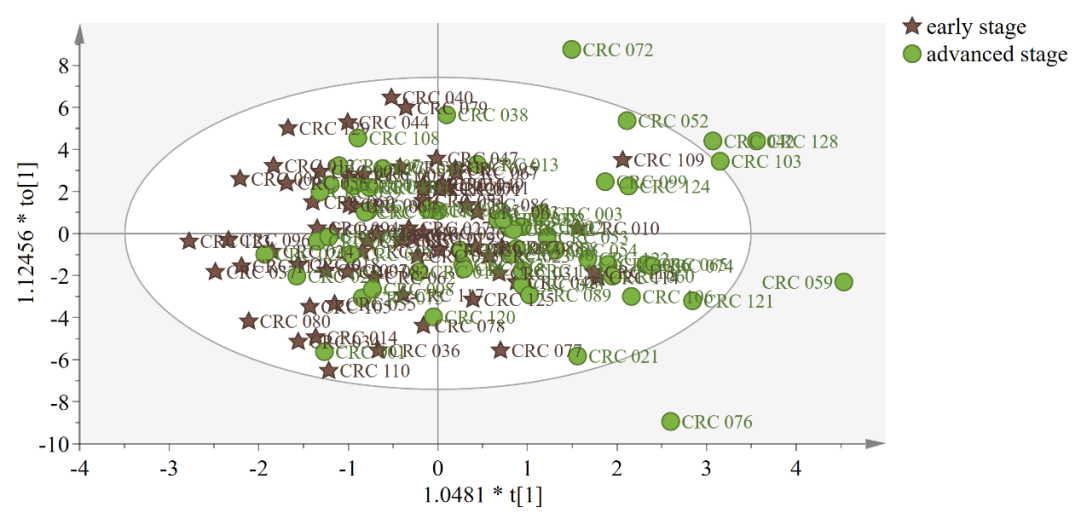


**Additional Fig. S5B**: Score scatter plot for OPLS-DA model of amino acids profiles in early and advanced stage CRC patients’ plasma samples.


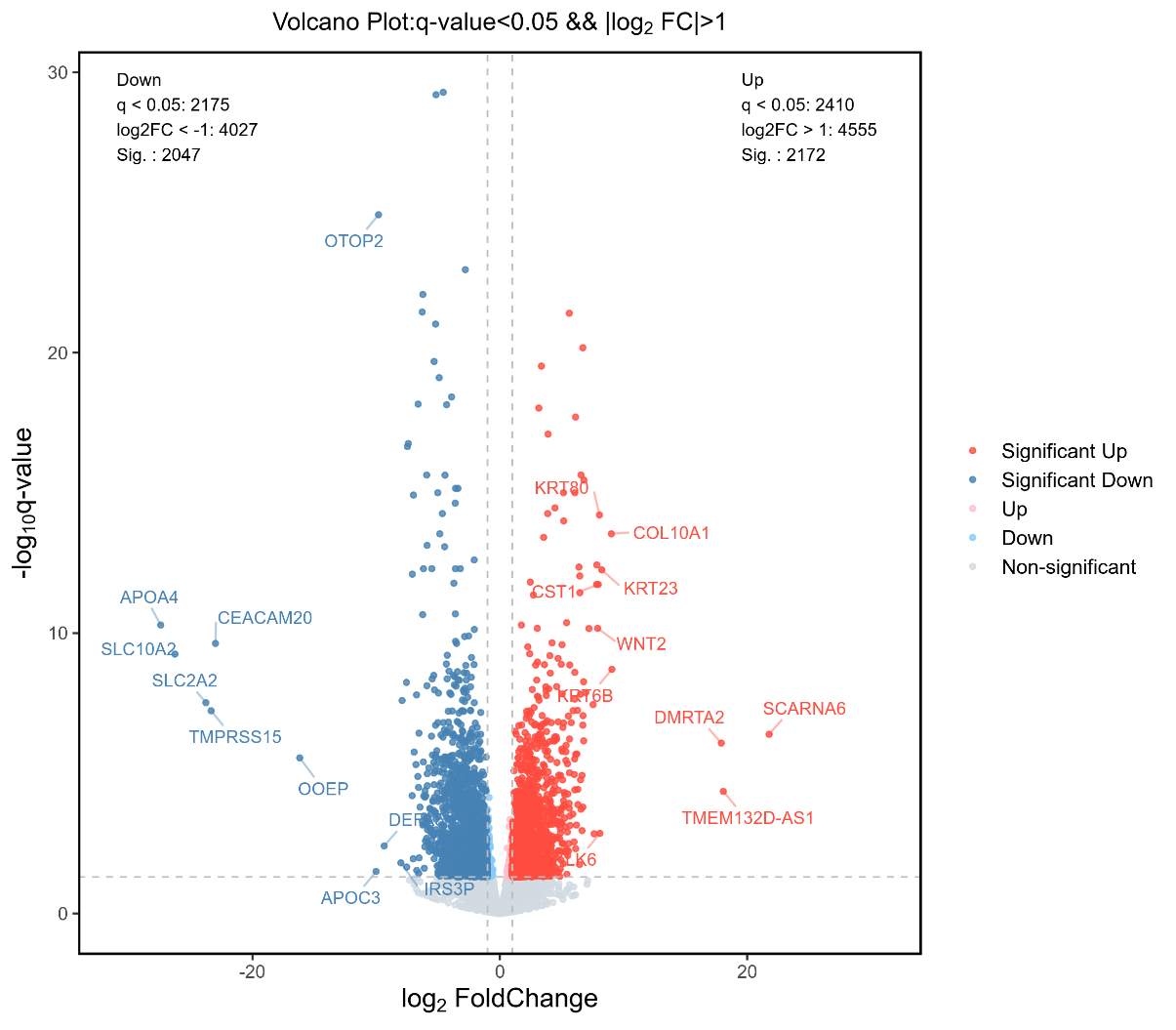


**Additional Fig. S6A** Volcano plot of differentially expressed genes in dataset GSE164541.


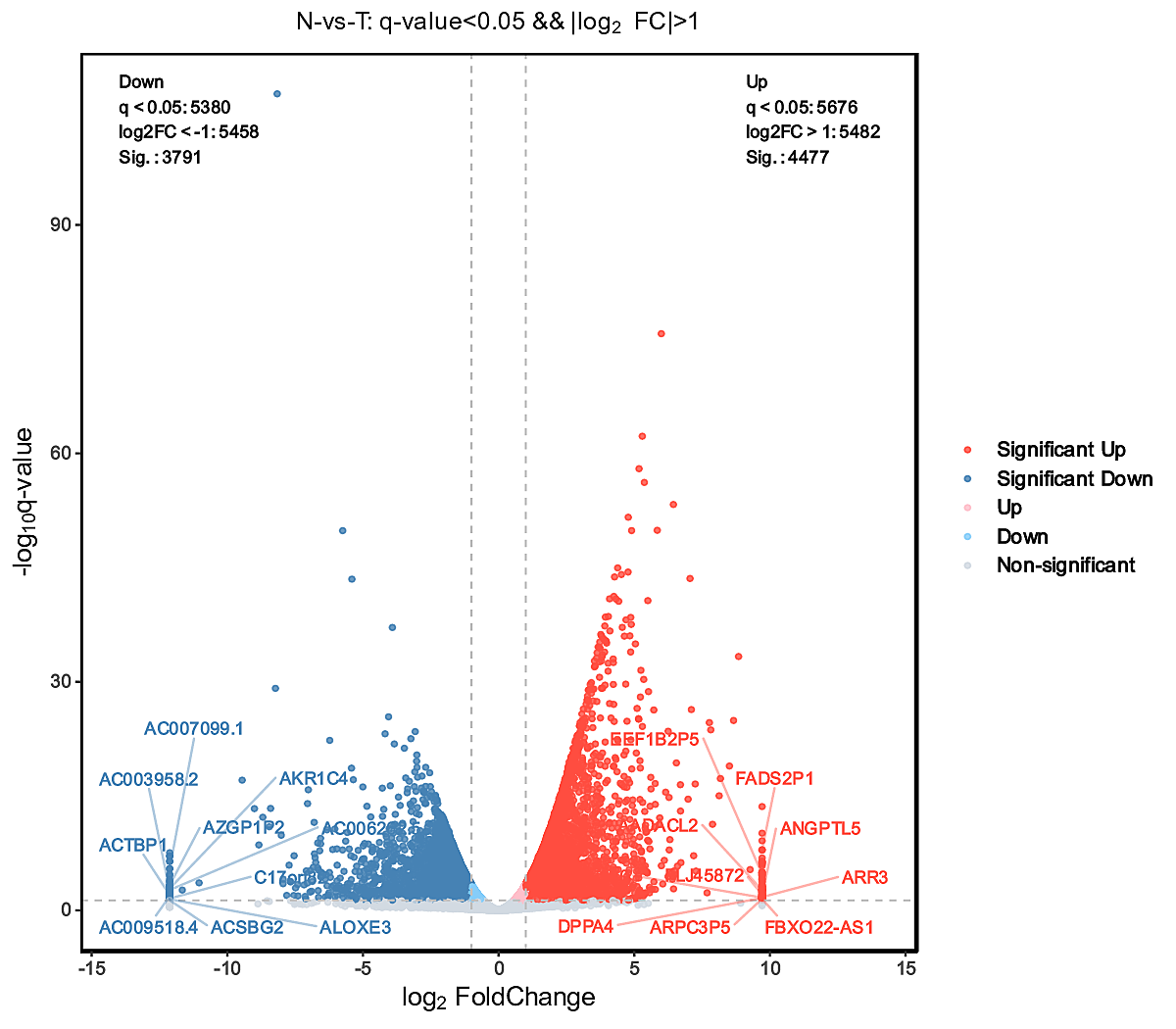


**Additional Fig.** S6B Volcano plot of differentially expressed genes in dataset GSE 138202.

| A | 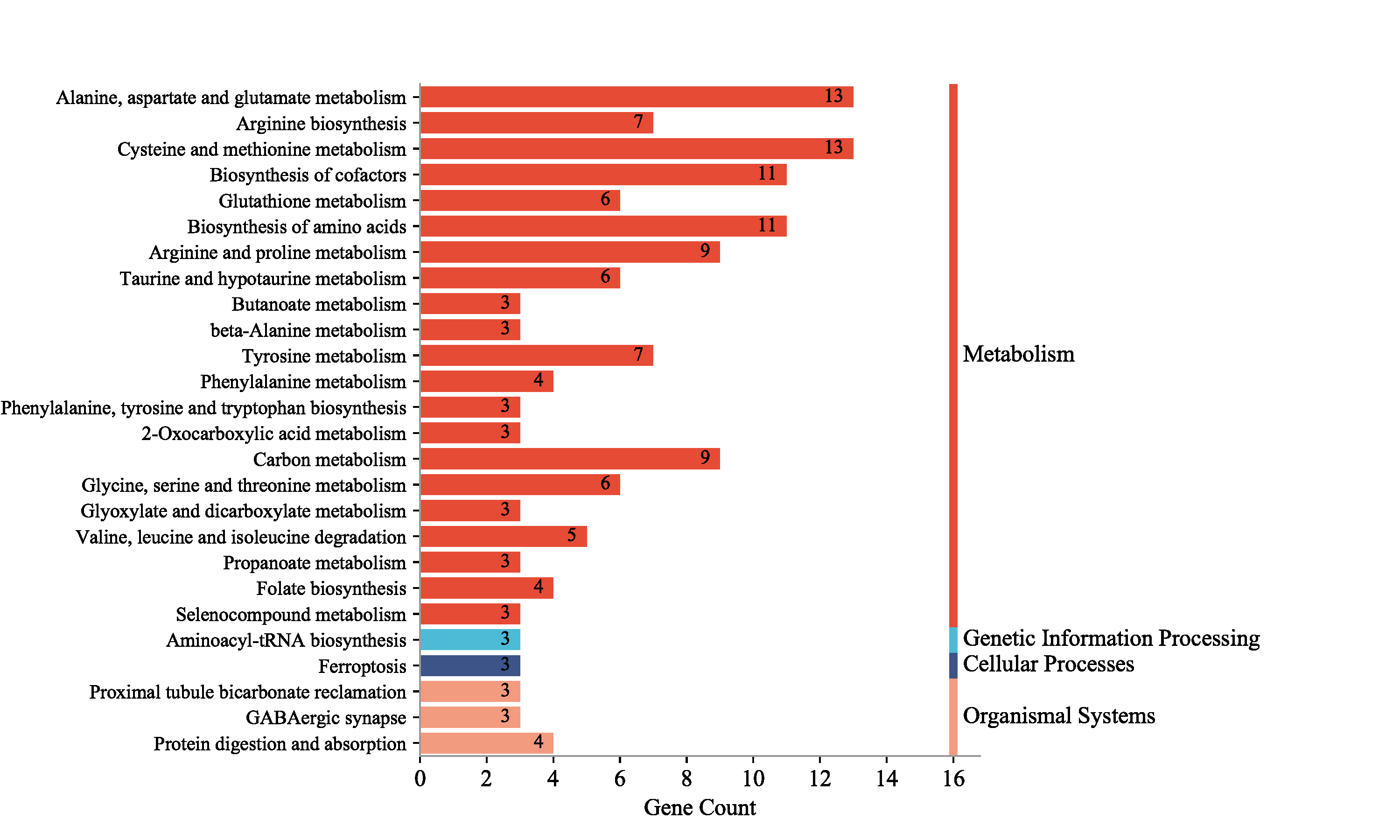 |
| --- | --- |
| B | 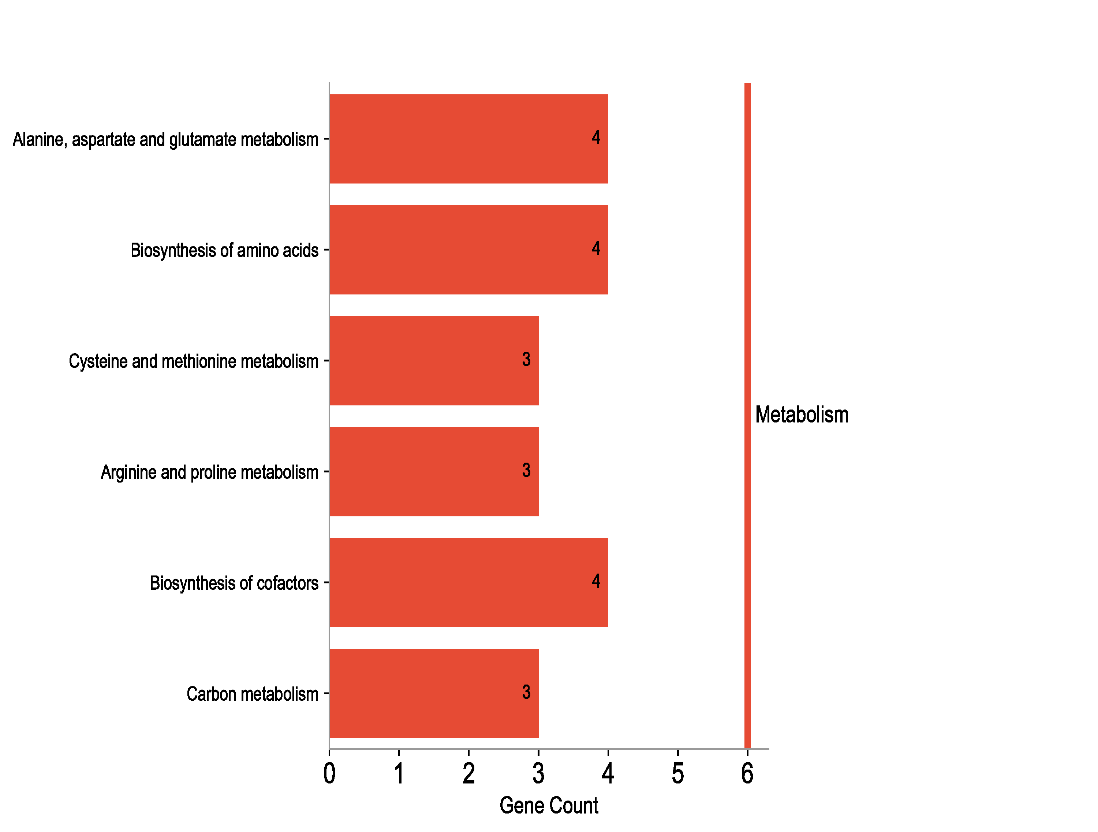 |

**Additional Fig. S7** Summarized pathway changes in tumorigenesis based on The KEGG pathway analysis. A: pathway enrichment based on GSE164541; B pathway enrichment based on GSE138202.

**Additional Table S1** Logistics regression model parameters

|  | **B value** | **Standard error** | **Wals value** | **df value** | **P value** | **Exp(B) value** | **Exp(B) 95% confidence interval** | |
| --- | --- | --- | --- | --- | --- | --- | --- | --- |
|  |  |  |  |  |  |  | **The lower limit** | **The upper limit** |
| Trp | 0.001 | 0.000 | 34.034 | 1 | 0.000 | 1.001 | 1.001 | 1.001 |
| Sar | 0.029 | 0.005 | 29.559 | 1 | 0.000 | 1.029 | 1.019 | 1.040 |
| Glu | -0.002 | 0.000 | 52.036 | 1 | 0.000 | 0.998 | 0.998 | 0.999 |
| Constant | -9.427 | 1.720 | 30.025 | 1 | 0.000 | 0.000 | - | - |

Note：“-” for null.

**Additional Table S2** Areas under the curve of diagnostic factors

| **Variable** | **AUC** | **Standard error** | **Asymptotic significance** | **Asymptotic 95% confidence interval** | |
| --- | --- | --- | --- | --- | --- |
|  |  |  |  | **The lower limit** | **The upper limit** |
| Trp | 0.861 | 0.022 | 0.000 | 0.818 | 0.903 |
| Sar | 0.888 | 0.019 | 0.000 | 0.852 | 0.925 |
| Glu | 0.896 | 0.016 | 0.000 | 0.864 | 0.928 |
| CEA | 0.809 | 0.023 | 0.000 | 0.764 | 0.853 |
| AFP | 0.402 | 0.031 | 0.002 | 0.341 | 0.462 |
| Joint factor  diagnostic model | 0.980 | 0.007 | 0.000 | 0.965 | 0.994 |

**Additional Table S3** The value of joint factor diagnostic model in the diagnosis of CRC

| **Joint factor diagnostic model diagnosis** | **Gold standard diagnosis** | | | | **Total** |
| --- | --- | --- | --- | --- | --- |
|  | **Positive （CRC patient）** | **Negative（Non-cancerous patient）** | | |  |
|  |  | **PC patient** | **HV** | **subtotal** |  |
| Positive | 205 | 2 | 13 | 15 | 220 |
| Negative | 41 | 31 | 398 | 429 | 470 |
| Total | 246 | 33 | 411 | 444 | 690 |

**Additional Table S4** The value of CEA in the diagnosis of CRC

| **CEA diagnosis** | **Gold standard diagnosis** | | | | **Total** |
| --- | --- | --- | --- | --- | --- |
|  | **Positive （CRC patient）** | **Negative（Non-cancerous patient）** | | |  |
|  |  | **PC patient** | **PC patient** | **PC patient** |  |
| Positive | 82 | 1 | 31 | 32 | 114 |
| Negative | 164 | 32 | 380 | 412 | 576 |
| Total | 246 | 33 | 411 | 444 | 690 |
